# Rapid Complexation of Aptamers by Their Specific Antidotes

**DOI:** 10.3390/molecules22060954

**Published:** 2017-06-08

**Authors:** Heidi Stoll, Heidrun Steinle, Nadja Wilhelm, Ludmilla Hann, Silju-John Kunnakattu, Miwako Narita, Christian Schlensak, Hans P. Wendel, Meltem Avci-Adali

**Affiliations:** 1 Department of Thoracic and Cardiovascular Surgery, University Hospital Tuebingen, 72076 Tuebingen, Germany; stollheidi@aol.de (H.Stoll); heidi.steinle@googlemail.com (H.Steinle); NL1282@gmx.de (N.W.); ludmilla.hann@klinikum.uni-tuebingen.de (L.H.); silju_j1984@yahoo.de (S.-J.K.); christian.schlensak@med.uni-tuebingen.de (C.S.); hans-peter.wendel@med.uni-tuebingen.de (H.P.W.); 2 Laboratory of Hematology and Oncology, Graduate School of Health Sciences, Niigata University, Niigata 951-8518, Japan; naritami@clg.niigata-u.ac.jp

**Keywords:** aptamers, antidote, therapeutics, complexation

## Abstract

Nucleic acid ligands, aptamers, harbor the unique characteristics of small molecules and antibodies. The specificity and high affinity of aptamers enable their binding to different targets, such as small molecules, proteins, or cells. Chemical modifications of aptamers allow increased bioavailability. A further great benefit of aptamers is the antidote (AD)-mediated controllability of their effect. In this study, the AD-mediated complexation and neutralization of the thrombin binding aptamer NU172 and Toll-like receptor 9 (TLR9) binding R10-60 aptamer were determined. Thereby, the required time for the generation of aptamer/AD-complexes was analyzed at 37 °C in human serum using gel electrophoresis. Afterwards, the blocking of aptamers’ effects was analyzed by determining the activated clotting time (ACT) in the case of the NU172 aptamer, or the expression of immune activation related genes *IFN-1β*, *IL-6*, *CXCL-10*, and *IL-1β* in the case of the R10-60 aptamer. Gel electrophoresis analyses demonstrated the rapid complexation of the NU172 and R10-60 aptamers by complementary AD binding after just 2 min of incubation in human serum. A rapid neutralization of anticoagulant activity of NU172 was also demonstrated in fresh human whole blood 5 min after addition of AD. Furthermore, the TLR9-mediated activation of PMDC05 cells was interrupted after the addition of the R10-60 AD. Using these two different aptamers, the rapid antagonizability of the aptamers was demonstrated in different environments; whole blood containing numerous proteins, cells, and different small molecules, serum, or cell culture media. Thus, nucleic acid ADs are promising molecules, which offer several possibilities for different in vivo applications, such as antagonizing aptamer-based drugs, immobilization, or delivery of oligonucleotides to defined locations.

## 1. Introduction

The use of controllable therapeutic agents and the blockage of their therapeutic effect after treatment can significantly reduce drug-induced side effects. Over the past 25 years, aptamers have gained clinical interest due to their several unique characteristics. Aptamers are short single-stranded oligonucleotides which can fold into three-dimensional structures. These molecules can bind with a high affinity and specificity to the target of interest [1]. The modifications of aptamers at defined positions enable the fine-tuning of their stability and bioavailability, and allow their application as theranostics [2]. 

The important criteria for the optimal application of drugs are the dosing accuracy and the controllability of their effect. Therefore, so-called antidotes (ADs) provide excellent possibilities, which can turn off the effect of administered drugs by competitive blocking, altering, binding, or general inactivation of the agents.

The binding of an aptamer to its target can be easily prevented by changing the three-dimensional structure of the aptamer, for example by addition of a complementary oligonucleotide which hybridizes to the aptamer. Thus, a further important characteristic of aptamers is that the binding ability of a specific aptamer can be abrogated by the addition of an oligonucleotide with a complementary sequence to the aptamer [3,4,5].

In this study, we analyzed the required time for the complexation of NU172 [6] and R10-60 [7,8] aptamers by their ADs in human serum. Against the concerns that the 3D folding of the aptamers and the existence of several proteins in biological fluids could impede the specific and quick recognition and binding of the ADs to the aptamers, here, we demonstrated using human serum the rapid binding of respective ADs to the NU172 or R10-60 aptamer and their complexation. Furthermore, the abolition of each aptamer´s effect was demonstrated in human blood or cell culture medium.

## 2. Materials and Methods

### 2.1. Oligonucleotides

The experiments were performed with two different DNA aptamers, the Toll-like receptor 9 (TLR 9) binding R10-60 aptamer and the thrombin binding NU172 aptamer, and their complementary DNA sequences, called ADs (Table 1). Furthermore, nonsense (NS) AD and nonsense aptamers were used as negative controls. All oligonucleotides were ordered as HPLC-purified from Ella Biotech GmbH (Martinsried, Germany).

### 2.2. Ethics Statement

The Ethics Committee of the University of Tuebingen approved the blood sampling procedures and all subjects gave written informed consent.

### 2.3. Generation of Human Serum

Human blood samples (7.5 mL) were collected in tubes containing kaolin-coated granulate (Sarstedt AG & Co., Nümbrecht, Germany) as a clot activator and the blood was incubated for approximately 30 min at room temperature. Then, the tubes were centrifuged for 15 min at 2000× *g*. The liquid supernatant (serum) was collected and frozen at −20 °C.

### 2.4. Collection of Human Blood for Determining of ACT (Activated Clotting Time)

A total volume of 4.5 mL blood was collected from the antecubital vein of each healthy, non-medicated volunteer into neutral monovettes or 3 IU/mL sodium heparin-containing (Ratiopharm, Ulm, Germany) monovettes.

### 2.5. Cultivation of PMDC05 Cells

The leukemic plasmacytoid dendritic cell line (PMDC05) is similar to normal plasmacytoid dendritic cells (pDCs) in its surface expression of phenotypic markers and expression of TLRs. These cells were donated from the Laboratory of Hematology and Oncology of Niigata University in Japan. PMDC05 cells were cultured in suspension culture flasks in IMDM medium (Iscove’s Modified Dulbecco’s Medium) containing 10% FBS (fetal bovine serum), 100 units/mL penicillin, and 100 μg/mL streptomycin at 37 °C, 5% CO_2_, and 20% O_2_. All cell culture reagents were obtained from Life Technologies (Darmstadt, Germany). A medium change was performed every 3 days. The number of cells was determined using a CASY^®^ cell counter (Roche Innovatis AG, Mannheim, Germany) and the cultivation was performed with 30 × 10^6^ cells/30 mL medium. 

### 2.6. Required Time for Binding of AD to Aptamers R10-60 and NU172 in Human Serum

Aptamers, respective ADs, and nonsense ADs or aptamers (10 μM) were denatured at 95 °C and cooled down to 37 °C to induce the three-dimensional folding of aptamers and ADs. The incubation of the aptamers with their respective AD was performed in 200 μL human serum at a molar ratio of 1:1 (1 μg/1 μg) at 37 °C and with shaking at 300 rpm for 2, 5, 10, 15, and 30 min. Nonsense controls were analyzed after 2 and 5 min of incubation. After each incubation time, samples were purified by phenol-chloroform precipitation and concentrated using Amicon Ultra 0.5 mL 10K centrifugal filters (Merck Millipore, Darmstadt, Germany) according to the manufacturer´s instructions. All steps were performed at 37 °C.

The aptamer/AD complexes were visualized using nondenaturing polyacrylamide gel electrophoresis (PAGE). Therefore, 400 ng of aptamers (R10-60, NU172) and their corresponding FAM (fluorescein amidite)-labeled AD, and nonsense controls were loaded onto gels, and 20 μL of concentrated samples after serum incubation was run on 10% nondenaturing polyacrylamide gel. Gels were first visualized on an UV transilluminator to detect the FAM-labeled AD or AD_NS and then stained with GelRed™ (Biotium Inc., Hayward, CA, USA) to detect all oligonucleotides. Subsequently, gels were documented using the gel documentation system (Gel Doc XR, Bio-Rad laboratory GmbH, Munich, Germany). The images were colored and overlaid using Adobe Photoshop Elements 14.0.

### 2.7. Generation of Aptamer/AD Complexes in Human Whole Blood

To fold oligonucleotides into their unique three-dimensional structures, aptamers, respective AD, and the controls, AD_NS or aptamer (NS), (10 μM) were denatured at 95 °C and then cooled down to 37 °C. The incubation of aptamers with their respective AD was performed in 1 mL human neutral blood at a molar ratio of 1:1 (5 μg/5 μg) at 37 °C. After the addition of the aptamer or NS aptamer, the blood was incubated with shaking at 300 rpm for 2 min. Thereafter, AD or AD_NS was added to the blood. After 2 min of incubation with shaking at 300 rpm, serum was generated using kaolin-coated granulate (Sarstedt AG & Co., Nümbrecht, Germany) as a clot activator. Samples were purified using phenol-chloroform precipitation and concentrated using Amicon Ultra 0.5 mL 10K centrifugal filters (Merck Millipore, Darmstadt, Germany) according to the manufacturer´s instructions. All steps were performed at 37 °C.

The generated aptamer/AD complexes were visualized using nondenaturing PAGE. For this, 400 ng of the aptamers (R10-60, NU172) and their corresponding FAM-labeled AD, and nonsense controls were loaded onto gels, and 10 μL of concentrated samples was run on 10% nondenaturing polyacrylamide gel. Firstly, FAM-labeled AD or AD_NS in the samples was visualized on an UV transilluminator and then stained with GelRed™ (Biotium Inc., Hayward, CA, USA) to detect all oligonucleotides. Gels were documented using the gel documentation system (Gel Doc XR, Bio-Rad laboratory GmbH, Munich, Germany). The images were colored and overlaid using Adobe Photoshop Elements 14.0.

### 2.8. Blocking of Aptamers by Generation of Aptamer/AD Complexes

#### 2.8.1. Determination of ACT of Blood after Addition of Thrombin Aptamer AD (NU172 (AD)) to NU172

To monitor the functional inhibition time of NU172 by its AD, ACT was determined after addition of 0.5 or 1 μM NU172 aptamer alone, as well as after the addition of 1 or 0.5 μM NU172 (AD) to the 1 μM NU172-containing human blood. The ACT of blood without oligonucleotides was measured as a negative control and blood containing heparin served as a positive control. Additionally, the ACT of blood containing 1 μM NU172 (AD_NS), 1 μM NU172 (NS), 1 μM NU172 (AD), or 1 μM NU172 (NS) and 1 μM NU172 (AD), or 1 μM NU172 and 1 μM NU172 (AD_NS) was analyzed.

Measurements were performed using a Hemochron Jr. II (International Technidyne Corporation, Edison, NJ, USA). This microcoagulation system utilizes a mechanical endpoint clotting mechanism which is monitored optically. Thereby, the ACT of whole blood samples can be measured within disposable test cuvettes. Hemochron^®^ Jr. High Range ACT (ACT+) cuvettes were used for the detection of the ACT of blood samples. After warming of the cuvettes in the system, samples were transferred onto the test channel and the clotting time was measured in seconds. 

After the withdrawal of venous blood, blood from neutral monovettes was incubated for 2 min with or without oligonucleotides. Heparin containing blood was respectively incubated for 2 min without addition of aptamers. Subsequently, the measurement of the ACT was performed at different times: 0 min (T0), after 5 min (T5), 15 min (T15), and 30 min (T30). Therefore, 50 μL of samples was transferred into the test cuvettes. 

To determine the clotting time, neutral blood (without heparin) was spiked with 0.5 and 1 μM NU172 aptamer or 1 μM of each oligonucleotide. After 2 min of incubation, 50 μL of each sample was measured at T0, T5, T15, and T30. Furthermore, the ACT of blood samples was measured after the addition of 0.5 μM and 1 μM NU172 (AD) or after the addition of 1 μM NU172 (AD_NS) to 1 μM NU172 containing neutral blood samples, as well as after the addition of 1 μM NU172 (AD) to 1 μM NU172 (NS)-containing neutral blood, as described above. All experiments were performed at 37 °C with blood samples from 3 donors (*n* = 3).

#### 2.8.2. Investigation of the Interruption of R10-60 Binding to TLR9 by Antidote Addition

In our previous study, we demonstrated that the recognition of the aptamer R10-60 by TLR9 in PMDC05 cells leads to the expression of pro-inflammatory cytokines [9], e.g., IFN-1β, IL-6, CXCL-10, and IL-1β. Thus, we determined the neutralization of the R10-60 aptamer binding ability to the TLR9 by the addition of R10-60 (AD) to the PMDC05 cells and subsequently analyzing the expression of IFN-1β, IL-6, CXCL-10, and IL-1β.

### 2.9. Incubation of PMDC05 Cells with R10-60 Aptamer and R10-60 (AD)

PMDC05 cells were incubated in 48-well suspension plates without and with addition of 10 μM R10-60 aptamer, R10-60 (AD), or R10-60 (AD_NS) alone, or with 10 μM R10-60 aptamer and 10 μM R10-60 (AD) or 10 μM R10-60 (AD_NS), for 4 h at 37 °C. Additionally, cells were incubated for 2 min with 10 μM R10-60 aptamer and then R10-60 (AD) was added to the PMDC05 medium and incubated also for 4 h at 37 °C. A PMDC05 cell suspension of 450 μL containing 1 × 10^6^ cells was used per well of a 48-well plate.

### 2.10. Isolation of Total RNA from PMDC05 Cells and Synthesis of cDNA

After 4 h of incubation, total RNA was isolated from PMDC05 cells using an Aurum™ Total RNA Mini Kit (BioRad Laboratories, Munich, Germany) according to the manufacturer’s instructions. The RNA concentration was measured using a BioPhotometer (Eppendorf, Hamburg, Germany). To perform real-time quantitative reverse transcription polymerase chain reaction (qRT-PCR) analyses, 300 ng RNA of each sample was transcribed using an iScript^TM^ cDNA synthesis kit (Bio-Rad Laboratories, Munich, Germany) into cDNA. Reverse transcription was performed under the following conditions: 5 min at 25 °C, 30 min at 42 °C, and 5 min at 85 °C. Synthesized cDNA was diluted 1:10 for qRT-PCR.

### 2.11. qRT-PCR

The analysis of IFN-1β, IL-6, CXCL-10, and IL-1β mRNA expression in samples was performed using iQ SYBR Green Supermix (BioRad Laboratories, Munich, Germany) according to the manufacturer´s recommendations. All qRT-PCR reactions were performed in triplicate in a CFX Connect™ Real-Time PCR Detection System (BioRad Laboratories, Munich, Germany). Expression of the constitutively expressed gene *GAPDH* (glyceraldehyde 3-phosphate dehydrogenase) was used as an internal control for RNA input. The primers used for the specific amplification of transcripts are listed in Table 2 and were ordered from Ella Biotech (Martinsried, Germany). PCR cycling conditions consisted of an initial hot start at 95 °C for 3 min to activate the hot-start iTaq DNA polymerase and to denature the template DNA, followed by 40 cycles of denaturing at 95 °C for 15 s, annealing at 63 °C for 30 s and elongation at 72 °C for 10 s. At the end of amplification process, melt-curve analysis was performed consisting of 50 melt cycles, beginning at 70 °C to 95 °C with increments of 0.5 °C per cycle to control the specific length of the amplified product. All qRT-PCR data were collected and analyzed using the Bio-Rad CFX Manager^TM^ Software Version 3.0 (Bio-Rad Laboratories, Munich, Germany). Levels of mRNA were normalized to GAPDH and the results are shown relative to control mRNA levels in samples without oligonucleotide addition. 

### 2.12. Statistical Analysis

Data are shown as means ± standard deviation (SD) or means ± standard error of the mean (SEM). One-way analysis of variance (ANOVA) for repeated measures followed by the Bonferroni’s multiple comparison test was performed to compare the groups in qRT-PCR measurements. Two-way ANOVA followed by the Bonferroni’s multiple comparison test was used to compare the groups in ACT measurement experiments. All statistical analyses were performed double-tailed using GraphPad Prism version 5.01. Differences of *p* < 0.05 were considered statistically significant.

## 3. Results

### 3.1. Required Time for AD Binding to Aptamers R10-60 and NU172 in Human Serum

**Figure 1:**
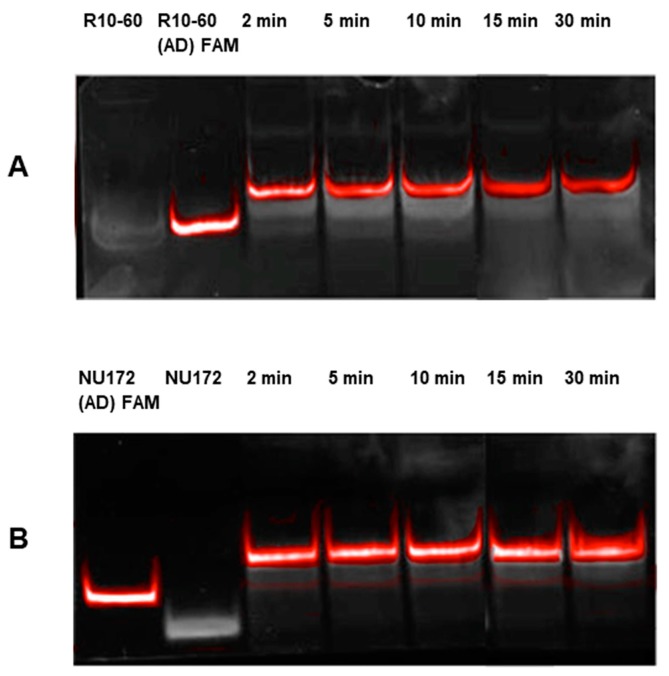
Determination of the time required for the formation of aptamer/antidote (AD) complexes using 10% nondenaturing polyacrylamide gel electrophoresis. Samples were analyzed after 2, 5, 10, 15, and 30 min of incubation in human serum. (**A**) Analyses of R10-60/R10-60 (AD) FAM complexes; (**B**) Analyses of NU172/NU172 (AD) FAM complexes. Firstly, FAM-labeled ADs were detected using UV-transilluminator (red colored bands) and then all oligonucleotides were detected using GelRed™ staining (white colored bands). An overlay of FAM and GelRed staining is presented.

To examine the required binding time of the FAM-labeled AD to their respective complementary sequences of R10-60 or NU172 aptamers, 1 μg aptamer and 1 μg (AD) FAM were incubated for 2, 5, 10, 15, and 30 min at 37 °C in human serum. After different times, the generated complexes were visualized on a 10% nondenaturing polyacrylamide gel. As shown in Figure 1. Already after 2 min, the aptamer/AD complexes were generated. The formation of the complexes was visualized by using FAM-labeled AD. The generation of complexes resulted in the shift of the band toward a higher molecular weight, since complexes migrate more slowly than the aptamer or AD alone. Only a slight amount of R10-60 aptamer and NU172 (AD) FAM remained unbound. However, complete neutralization of aptamers can be achieved by using optimized concentrations of AD and aptamer. Furthermore, after 2 min, the generated complexes remained neutralized up to 30 min.

**Figure 2:**
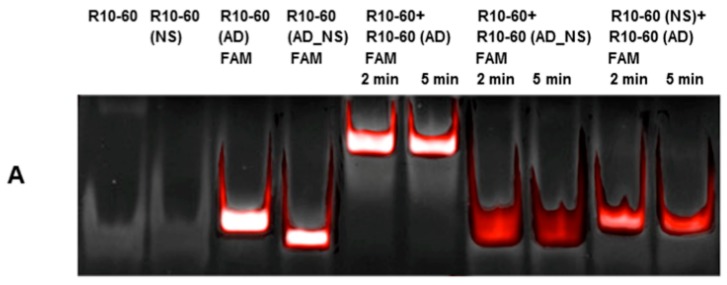

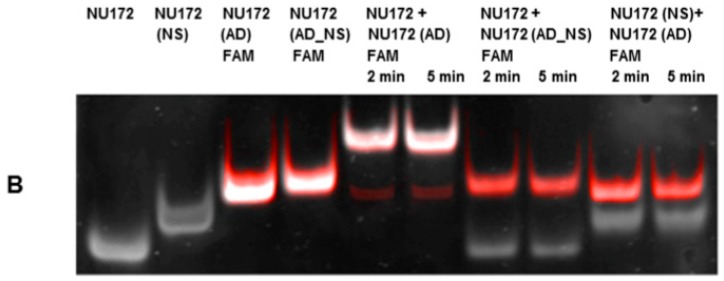
Analyses of specific binding of antidotes (ADs) to their target aptamers and the formation of aptamer/AD complexes using 10% nondenaturing polyacrylamide gel electrophoresis. Samples were analyzed after 2 and 5 min of incubation in human serum. (**A**) Analyses of R10-60 (AD_NS) FAM binding to the R10-60 aptamer and the R10-60 (NS) aptamer binding to R10-60 (AD) FAM; (**B**) Analyses of NU172 (AD_NS) FAM binding to the NU172 aptamer and the NU172 (NS) aptamer binding to NU172 (AD) FAM. Firstly, FAM-labeled ADs were detected using an UV-transilluminator (red colored bands) and then all oligonucleotides were detected using GelRed™ staining (white colored bands). An overlay of FAM and GelRed staining is presented.

To demonstrate the specific binding of the ADs to their respective aptamer, binding experiments were repeated with nonsense aptamers (R10-60 (NS) and NU172 (NS)) and FAM-labeled nonsense AD (R10-60 (AD_NS) and NU172 (AD_NS)). For this, 1 μg of aptamer or nonsense aptamer and 1 μg of AD or nonsense AD were incubated for 2 and 5 min at 37 °C in serum. The generated complexes were visualized on a 10% nondenaturing polyacrylamide gel. As shown in Figure 2, only the incubation with the respective AD resulted in generation of aptamer/AD complexes, which can be seen by the shift of the bands toward higher molecular weight. The complexes demonstrated slower migration compared to the aptamer or AD alone. The formation of complexes was visualized by using FAM-labeled AD and AD_NS.

### 3.2. Generation of Aptamer/AD Complexes in Whole Human Blood

**Figure 3:**
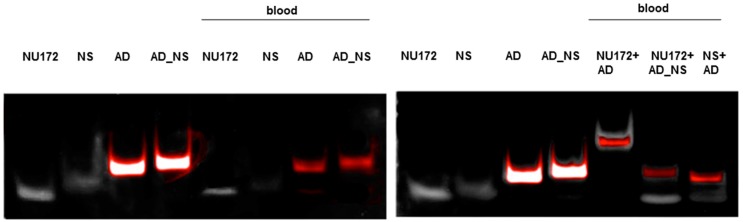
Analyses of the specific binding of the NU172 antidote (AD) to the NU172 aptamer in whole human blood and the formation of aptamer/AD complexes using 10% nondenaturing polyacrylamide gel electrophoresis. Blood was first incubated for 2 min with 5 μg NU172. Subsequently, 5 μg NU172 (AD) was added and incubated for 2 min. After isolation of oligonucleotides, samples of 10 μL were analyzed. NS and AD_NS were used as negative controls. Firstly, FAM-labeled ADs were detected using an UV-transilluminator (red colored bands) and then all oligonucleotides were detected using GelRed™ staining (white colored bands). An overlay of FAM and GelRed staining is presented. NS: NU172 (NS), AD: NU172 (AD) FAM, AD_NS: NU172 (AD_NS) FAM.

**Figure 4:**
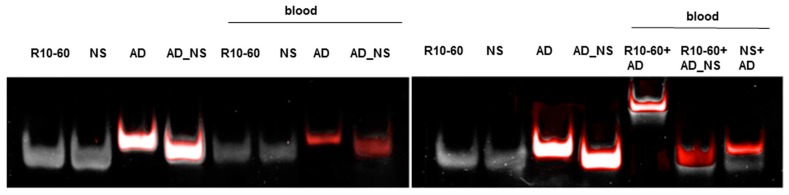
Analyses of specific binding of R10-60 antidote (AD) to the R10-60 aptamer in whole human blood and the formation of aptamer/AD complexes using 10% nondenaturing polyacrylamide gel electrophoresis. Blood was first incubated for 2 min with 5 μg R10-60. Subsequently, 5 μg R10-60 (AD) was added and incubated for 2 min. After isolation of oligonucleotides, samples of 10 μL were analyzed. NS and AD_NS were used as negative controls. Firstly, FAM-labeled ADs were detected using an UV-transilluminator (red colored bands) and then all oligonucleotides were detected using GelRed™ staining (white colored bands). An overlay of FAM and GelRed staining is presented. NS: R10-60 (NS), AD: R10-60 (AD) FAM, AD_NS: R10-60 (AD_NS) FAM.

The ability of NU172 (AD) to specifically bind to the NU172 aptamer in human whole blood was demonstrated in Figure 3. For this, 5 μg NU172 was added to 1 mL human blood and incubated for 2 min at 37 °C. Afterwards, 5 μg NU172 (AD) FAM was added and after 2 min of incubation, the oligonucleotides were purified and analyzed by 10% nondenaturing PAGE. The same experiment was performed with R10-60 (AD) and the R10-60 aptamer in human whole blood (Figure 4). The incubation of aptamer-containing human blood with the AD also resulted in the generation of aptamer/AD complexes with a higher molecular weight. In contrast, ADs were not able to bind the NS aptamers, and AD_NS could not bind to the aptamers.

### 3.3. Abrogation of the Aptamer Binding to the Target by Generation of Aptamer/AD Complexes

#### 3.3.1. Determination of the ACT of Blood after Addition of Thrombin Aptamer AD (NU172 (AD)) to NU172

**Figure 5:**
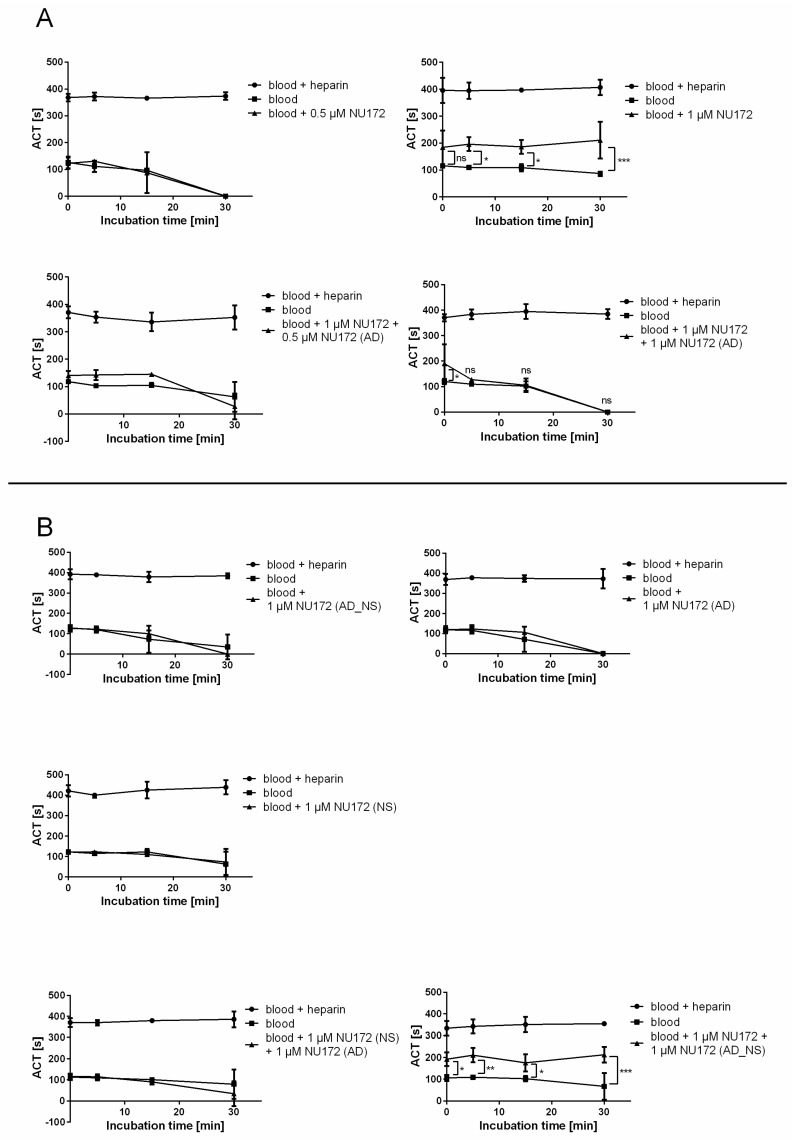
Measurement of the activated clotting time (ACT) of blood samples with different additives. (**A**) 0.5 μM or 1 μM NU172, 1 μM NU172 and 0.5 or 1 μM NU172 (AD), (**B**) 1 μM NU172 (AD_NS), 1 μM NU172 (NS), 1 μM NU172 (AD), or 1 μM NU172 (NS) and 1 μM NU172 (AD), or 1 μM NU172 and 1 μM NU172 (AD_NS). Blood without anticoagulants was used as a negative control and blood with heparin was used as a positive control. Differences were assessed by two-way ANOVA. Results are expressed as mean ± SD (*n* = 3) (* *p* < 0.05, ** *p* < 0.01, *** *p* < 0.001).

The time to abrogate the anticoagulant effect of NU172 was determined by the addition of NU172 (AD) to the blood samples. For this, neutral blood (negative control), blood containing heparin (positive control), and blood containing 0.5 or 1 μM NU172 aptamer alone or with the addition of 0.5 or 1 μM NU172 (AD) to 1 μM NU172-containing blood were used. The ACT in seconds was determined after 0, 5, 15, and 30 min of incubation (Figure 5A). Furthermore, blood samples containing 1 μM NU172 (AD_NS), 1 μM NU172 (NS), 1 μM NU172 (AD), or 1 μM NU172 (NS) and 1 μM NU172 (AD), or 1 μM NU172 and 1 μM NU172(AD_NS) were analyzed (Figure 5B). All measurements were performed with three samples. The measurement of blood containing heparin displayed clotting at around 400 s, while neutral blood clotted quickly. The addition of 0.5 μM NU172 thrombin aptamer was not able to prolong the ACT, thus the concentration was increased to 1 μM (Figure 5A). The ACT was already increased after 2 min of incubation with human blood (T0), and after T5 a statistically significant increase in the ACT (196 ± 26 s) was determined, which indicated that the aptamer blocked the thrombin and its activation pathway. Until T30, the ACT remained significantly increased compared to the neutral blood. In contrast, the addition of 1 μM NU172 (AD), 1 μM NU172 (AD_NS), or 1 μM NU172 (NS) was not able to increase the ACT (Figure 5B). Blood containing NU172 (AD), NU172 (AD_NS), or NU172 (NS) showed a similar blood clotting behavior as the neutral blood without anticoagulants, since these oligonucleotides are not specific for thrombin. 

The addition of 1 μM NU172 (AD) to the blood samples containing 1 μM NU172 could quickly interrupt the binding of the aptamer to thrombin. After 5 min of AD addition, the ACT decreased to the level of neutral blood (Figure 5A). Although not significant, the addition of 0.5 μM NU172 (AD) to 1 μM NU172 containing blood resulted in a slightly higher ACT until 15 min, compared to the negative control (Figure 5A). This was probably caused by remaining excess of aptamers in the blood. Furthermore, NU172 could not be neutralized by using 1 μM NU172 (AD_NS) (Figure 5B) and the ACT remained significantly increased until 30 min. In contrast, 1 μM NU172 (NS) and the addition of 1 μM NU172 (AD) showed no influence on ACT (Figure 5B).

#### 3.3.2. Determination of the Abolishment of R10-60 Binding to TLR9 after Addition of R10-60 (AD)

**Figure 6:**
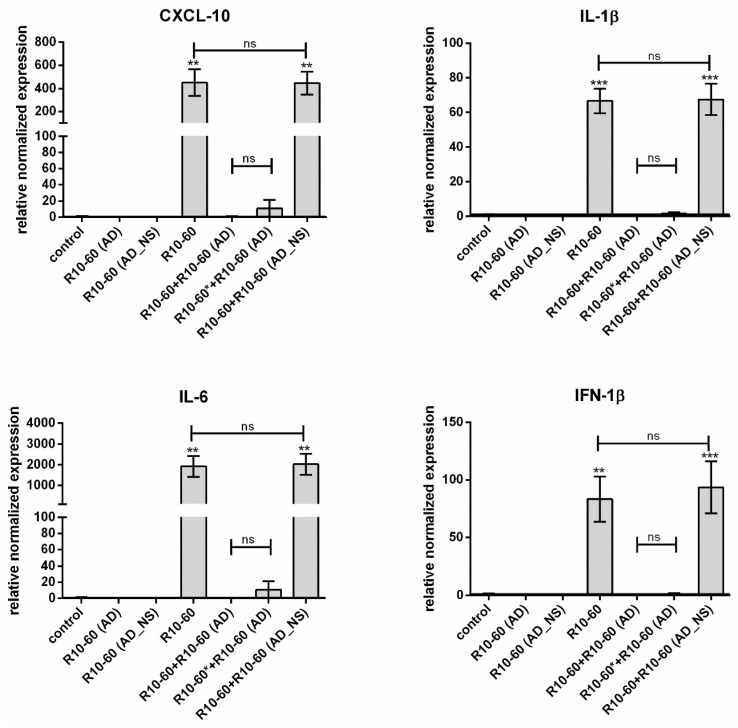
Blocking of R10-60 induced PMDC05 cell activation by adding of R10-60 (AD). IFN-1β, CXCL-10, IL-1β, and IL-6 expression in PMDC05 cells was determined using qRT-PCR analyses after incubation of cells for 4 h without or with R10-60 aptamer, R10-60 (AD), or R10-60 (AD_NS) alone, or R10-60 aptamer and R10-60 (AD) or R10-60 (AD_NS) together. The sample marked with * was incubated for 2 min with R10-60 and then R10-60 (AD) was added and incubated for 4 h at 37 °C. Differences were assessed by ANOVA for repeated measures. Results are expressed as mean ± SEM (*n* = 3) (** *p* < 0.01, *** *p* < 0.001).

PMDC05 cells were incubated for 4 h at 37 °C with 10 μM R10-60, R10-60 (AD), or R10-60 (AD_NS) alone, or with 10 μM R10-60 and 10 μM R10-60 (AD) or 10 μM R10-60 (AD_NS) together. Additionally, cells were first incubated for 2 min with 10 μM R10-60 aptamer and then R10-60 (AD) was added to the PMDC05 medium and incubated also for 4 h. This sample is marked with *. After 4 h, qRT-PCR analyses were performed to determine TLR9-mediated activation of PMDC05 cells, which leads to the up-regulation of IFN-1β, CXCL-10, IL-1β, and IL-6 expression. The stimulation of PMDC05 cells with 10 μM R10-60 resulted in approximately 450-fold upregulation of CXCL-10, 67-fold upregulation of IL-1β, 1921-fold upregulation of IL-6, and 83-fold upregulation of IFN-1β (Figure 6). However, the stimulation of cells was prevented by the addition of R10-60 (AD) to the R10-60-containing cell suspension. The addition of R10-60 (AD) after 2 min incubation of cells with R10-60 showed an increase in the expression of CXCL-10 and IL-6, however, this was not significant. In contrast, the addition of R10-60 (AD_NS) to R10-60 was not able to prevent the activation of PDMC05 cells, since R10-60 could not be complexed by R10-60 (AD_NS). Furthermore, R10-60 (AD) and R10-60 (AD_NS) were not able to activate TLR9.

## 4. Discussion

The intake of medicines is also associated with unwanted side effects. Thus, the interest in the development of new drugs with reduced side effects and targeted drug delivery has increased in recent years. Aptamers, having high binding specificity and affinity to specific targets, such as cells or proteins (growth factors, transcription factors, enzymes, immunoglobulins, and receptors), and the ability to modulate the targets’ activities, represent an interesting class of drugs. The high binding affinities and the option to use a complementary oligonucleotide as an AD for the regulation or neutralization of aptamer activity [4,10,11] is a great advantage of these DNA-based drugs. The binding of an AD to the aptamer can disrupt the less stable tertiary structure of the aptamer by the formation of more thermodynamically stable Watson–Crick base-pairing [12].

In this study, ADs against the thrombin binding aptamer NU172 (ARC2172) [6] and the TLR9 binding aptamer R10-60 [7,8] were used. The ability to abrogate the target binding and the inhibitory and activating effects of the aptamers was analyzed. Furthermore, the required time for the generation of double-stranded complexes was examined by gel electrophoresis analyses. 

Already after 2 min, aptamer/AD complexes were generated. Thereby, the ability of the AD to quickly bind to complementary sequences was demonstrated in human serum containing thousands of proteins with varying concentrations, wherein albumin and immunoglobulin G (IgG) account for more than 60% [13]. Furthermore, the anticoagulant effect of NU172 could be abrogated 5 min after the addition of 1 μM NU172 (AD) to 1 μM NU172 in human whole blood. Additionally, the immune stimulating effect of R10-60 was prevented by the addition of R10-60 (AD) to the PMDC05 cells. Thereby, ADs were able to efficiently bind to the complementary aptamer sequences without the need for denaturation of the oligonucleotides’ three-dimensional structure, for example by heat treatment or addition of denaturing agents. The sequence-specific binding of the ADs was demonstrated by using nonsense ADs or nonsense aptamers. However, it should also be mentioned that the complexation of aptamers in tissues could take a longer time when ADs are delivered intravenously. Nevertheless, to enable the rapid complexation of aptamers, an AD could be delivered site-specifically by local application or targeted delivery.

Oligonucleotides can enter the cells by nonspecific endocytosis as well as via interaction with different cell surface receptors, such as the receptor for advanced glycation end-products (RAGE) [14], DEC-205 [15], and macrophage scavenger receptors [16]. After the delivery of sequestered nucleic acids in endosomes, TLR9 can sense DNA molecules and lead to the production of inflammatory cytokines. In the case of R10-60, the exact mechanism of internalization is unknown, but it is suggested that cell surface receptors are involved [8]. In this study, we suppose that the R10-60 AD binds outside the cells to the R10-60 aptamers and forms complexes with them. Thereby, the potential interaction of R10-60 with receptors on the cell surface could be prevented, or, in the case of non-specific endocytosis, the endosome-sequestered R10-60 AD complexes could no longer bind and activate TLR9.

The kinetics of the AD binding to the aptamer can differ after the binding of the aptamer to its target. In this study, a rapid complexation of NU172 with NU172 (AD) was demonstrated in serum, which did not contain the coagulation factors. However, a rapid neutralization of the aptamer´s effect could also be shown in human blood containing the target, 5 min after the addition of the AD. In previous studies, Rusconi and colleagues also demonstrated that an AD to the FIXa RNA aptamer was able to neutralize the aptamer-induced anticoagulation rapidly (within 10 min) in plasma [3,4].

Furthermore, for the in vivo application of aptamer–AD pairs, especially in human blood, the nucleic acids should possess adequate stability to be effective. In contrast to unmodified RNA aptamers with a half-life of seconds, DNA aptamers are more stable, generally with a half-life of approximately 60 to 120 min [17,18]. However, the stability of aptamers can be further increased by the incorporation of modified nucleotides and 5′ end modifications, such as conjugation of polyethylene glycol (PEG) or capping of the 3′ end with an inverted deoxythymidine (dT) residue [19,20].

The results of this study demonstrated the rapid and specific binding of ADs to aptamers. Here, we selected two different aptamers with their ADs to demonstrate this interesting feature. Hereby, we aim to highlight that besides target binding, a further important advantage of aptamers is that their effect can be rapidly modulated or interrupted by a corresponding AD. 

## 5. Conclusions

In this study, we demonstrated the quick binding of ADs to their complementary single-stranded DNA molecules (aptamers) in human serum and blood, and in cell culture medium containing 10% FBS. The AD-based neutralization of the aptamer’s tertiary structure and thereby its function, enables the generation of controllable drugs. Furthermore, this phenomenon can also be used for other applications, e.g., to deliver drugs conjugated to aptamers to desired sites in vivo or to modulate the release of bound targets from aptamers. Thus, the application of non-coding nucleic acid pairs is a powerful tool for the modulation of cell and protein activities, delivery of cargos, and site-specific immobilization of desired molecules at defined positions. The findings of this study demonstrate the diverse application possibilities of aptamer-AD pairs.

## Figures and Tables

**Table 1:** List of used oligonucleotides.

Name	Length (Nucleotides)	Sequence of Oligonucleotides 5′ → 3′
**R10-60**	39	CCAGTCGTACAGGAAACATGCGTTCTAGATGTTCGGGGC
**R10-60 (NS)**	39	CATCAGTTACATGCACTATCAGTACTATCGATCATGCAT
**R10-60 (AD)**	39	GCCCCGAACATCTAGAACGCATGTTTCCTGTACGACTGG
**R10-60 (AD) FAM**	39	FAM-C18-GCCCCGAACATCTAGAACGCATGTTTCCTGTACGACTGG
**R10-60 (AD_NS)**	39	AGTACTGATAGTGCATGTAACTGATGCTTAGCATGCAAT
**R10-60 (AD_NS) FAM**	39	FAM-C18-AGTACTGATAGTGCATGTAACTGATGCTTAGCATGCAAT
**NU172**	26	CGCCTAGGTTGGGTAGGGTGGTGGCG
**NU172 (NS)**	26	CATCAGTTACATGCACTATCAGTACT
**NU172 (AD)**	26	CGCCACCACCCTACCCAACCTAGGCG
**NU172 (AD) FAM**	26	FAM-C18-CGCCACCACCCTACCCAACCTAGGCG
**NU172 (AD_NS)**	26	AGTACTGATAGTGCATGTAACTGATG
**NU172 (AD_NS) FAM**	26	FAM-C18-AGTACTGATAGTGCATGTAACTGATG

Abbreviations: AD: Antidote; FAM: Fluorescein amidite; NS: Nonsense.

**Table 2:** List of primers.

Genes	Forward Primer (5′ → 3′)	Reverse Primer (5′ → 3′)
***IFN-1β***	TACCTGAAGGCCAAGGAGTACAG	CGGAGGTAACCTGTAAGTCTGTTAA
***CXCL-10***	AAGTGGCATTCAAGGAGTACC	ACGTGGACAAAATTGGCTTGC
***IL-6***	CACACAGACAGCCACTCACCTC	CTGCCAGTGCCTCTTTGCTG
***IL-1β***	CCCACAGACCTTCCAGGAGA	CGGAGCGTGCAGTTCAGTG
***GAPDH***	TCAACAGCGACACCCACTCC	TGAGGTCCACCACCCTGTTG
